# Implementation of a Clinical Decision Support System for Antimicrobial Prescribing in Sub-Saharan Africa: Multisectoral Qualitative Study

**DOI:** 10.2196/45122

**Published:** 2024-10-07

**Authors:** Nathan Peiffer-Smadja, Sophie Descousse, Elsa Courrèges, Audrey Nganbou, Pauline Jeanmougin, Gabriel Birgand, Séverin Lénaud, Anne-Lise Beaumont, Claire Durand, Tristan Delory, Josselin Le Bel, Elisabeth Bouvet, Sylvie Lariven, Eric D'Ortenzio, Issa Konaté, Marielle Karine Bouyou-Akotet, Abdoul-Salam Ouedraogo, Gisèle Affoue Kouakou, Armel Poda, Corinne Akpovo, François-Xavier Lescure, Aristophane Tanon

**Affiliations:** 1 Université Paris Cité et Université Sorbonne Paris Nord Inserm IAME Paris France; 2 Antibioclic Steering Committee Paris France; 3 Infectious Diseases Department Bichat-Claude Bernard Hospital Assistance-Publique Hôpitaux de Paris Paris France; 4 CPias Centre Hospitalo-Universitaire de Nantes Nantes France; 5 CHU de Treichville Centre Hospitalo-universitaire de Treichville Abidjan Cote D'Ivoire; 6 Innovation and Clinical Research Unit Annecy-Genevois Hospital Epagny-Metz-Tessy France; 7 Department of General Practice Université Paris Diderot Université de Paris Paris France; 8 ANRS - Maladies infectieuses émergentes INSERM Paris France; 9 CHU Point G Bamako Mali; 10 Université des Sciences de la Santé Libreville Gabon; 11 CHU Bobo Dioulasso Bobo Dioulasso Burkina Faso; 12 CHU de Treichville Abidjan Cote D'Ivoire

**Keywords:** antimicrobial resistance, implementation research, Consolidated Framework for Implementation Research, CDSS, mobile health, mHealth, eHealth, mobile phone

## Abstract

**Background:**

Suboptimal use of antimicrobials is a driver of antimicrobial resistance in West Africa. Clinical decision support systems (CDSSs) can facilitate access to updated and reliable recommendations.

**Objective:**

This study aimed to assess contextual factors that could facilitate the implementation of a CDSS for antimicrobial prescribing in West Africa and Central Africa and to identify tailored implementation strategies.

**Methods:**

This qualitative study was conducted through 21 semistructured individual interviews via videoconference with health care professionals between September and December 2020. Participants were recruited using purposive sampling in a transnational capacity-building network for hospital preparedness in West Africa. The interview guide included multiple constructs derived from the Consolidated Framework for Implementation Research. Interviews were transcribed, and data were analyzed using thematic analysis.

**Results:**

The panel of participants included health practitioners (12/21, 57%), health actors trained in engineering (2/21, 10%), project managers (3/21, 14%), antimicrobial resistance research experts (2/21, 10%), a clinical microbiologist (1/21, 5%), and an anthropologist (1/21, 5%). Contextual factors influencing the implementation of eHealth tools existed at the individual, health care system, and national levels. At the individual level, the main challenge was to design a user-centered CDSS adapted to the prescriber’s clinical routine and structural constraints. Most of the participants stated that the CDSS should not only target physicians in academic hospitals who can use their network to disseminate the tool but also general practitioners, primary care nurses, midwives, and other health care workers who are the main prescribers of antimicrobials in rural areas of West Africa. The heterogeneity in antimicrobial prescribing training among prescribers was a significant challenge to the use of a common CDSS. At the country level, weak pharmaceutical regulations, the lack of official guidelines for antimicrobial prescribing, limited access to clinical microbiology laboratories, self-medication, and disparity in health care coverage lead to inappropriate antimicrobial use and could limit the implementation and diffusion of CDSS for antimicrobial prescribing. Participants emphasized the importance of building a solid eHealth ecosystem in their countries by establishing academic partnerships, developing physician networks, and involving diverse stakeholders to address challenges. Additional implementation strategies included conducting a local needs assessment, identifying early adopters, promoting network weaving, using implementation advisers, and creating a learning collaborative. Participants noted that a CDSS for antimicrobial prescribing could be a powerful tool for the development and dissemination of official guidelines for infectious diseases in West Africa.

**Conclusions:**

These results suggest that a CDSS for antimicrobial prescribing adapted for nonspecialized prescribers could have a role in improving clinical decisions. They also confirm the relevance of adopting a cross-disciplinary approach with participants from different backgrounds to assess contextual factors, including social, political, and economic determinants.

## Introduction

### Background

Antimicrobial resistance poses a global public health threat that affects both high-income countries and low- and middle-income countries (LMICs). In West African countries, the misuse and overuse of antimicrobials are further exacerbated by contextual factors, such as inappropriate antimicrobial prescribing practices, over-the-counter availability of antimicrobials, and low levels of public awareness. In addition, a specific socioeconomic context marked by disparities in health coverage, poor regulation in the pharmaceutical sector, and health care systems with limited resources also contribute to the misuse of antimicrobials [1]. Deviations from existing guidelines on antimicrobial use, including delays and overuse, are linked to both patient and health care professional behaviors [2,3].

Clinical decision support systems (CDSSs) could address the suboptimal use of antimicrobials by providing physicians with updated clinical practice guidelines and assisting them in prescribing appropriate antimicrobial treatments. For instance, ALMANACH, a CDSS designed for low-resource settings, has been shown to reduce by 80% the volume of antibiotics prescribed to children with acute illnesses in Tanzania [4]. Antibioclic [5], a not-for-profit CDSS developed by French university clinicians for antimicrobial prescribing in primary care, allows clinicians to obtain personalized recommendations for diagnostic and therapeutic management in just a few clicks, tailored to each clinical situation and patient characteristic [6,7].

A preimplementation study was carried out in Burkina Faso to assess the feasibility of adapting a CDSS for antimicrobial prescribing to the West African context [6]. In April 2020, Antibioclic Afrique was adapted from Antibioclic and co-designed to implement COVID-19 guidelines in various West African countries. It was codeveloped by clinicians from Ivory Coast, Gabon, France, Burkina Faso, Senegal, and Mali in partnership with the Antibioclic committee, the African Society for Infectious Diseases (SAPI), and the French Society for Infectious Diseases (SPILF). Antibioclic Afrique [8,9] is a Francophone nonprofit-oriented CDSS that is freely available on the web and as a smartphone app on iOS (Apple Inc) and Android (Google LLC). The recommendations used in the CDSS were derived from national clinical practice guidelines from the 5 partner countries or the World Health Organization (WHO) guidelines if national guidelines were not available and were gradually transformed into decision trees in Antibioclic Afrique. In a more illustrative manner, after the clinicians have made the diagnosis, the tool enables them to access specific pathologies during their consultation, such as “purulent meningitis,” and to input clinical elements related to the diagnosis. This input leads to a final page recommending the prescription of a particular type, duration, and dosage of antimicrobial tailored to the patient [7].

The implementation of a CDSS is influenced by the existing digital health environment at the national and global levels. In 2005, the WHO adopted a resolution establishing an eHealth strategy and recognized its role in achieving Millennium Development Goals, particularly in health system strengthening and extending universal health care coverage [10]. The WHO has developed several tools and guidelines to support and evaluate eHealth initiatives and provide technical assistance [11-13].

Concerning eHealth policies among the 5 partner countries, Côte d’Ivoire, Burkina Faso, Mali, and Senegal have developed strategies to develop eHealth and included it in national health development plans [14-16]. Some countries have a robust ecosystem of eHealth stakeholders and have quickly developed eHealth tools, including tools to fight COVID-19. For example, the Burkina Faso Ministry of Health developed 3 mobile phone apps to detect suspected COVID-19 cases, follow them up, and search for unreported contact cases [17]. Senegal developed the DAANCOVID19 initiative to create and share relevant content related to COVID-19 through social media. Moreover, a special committee is responsible for designing innovative eHealth solutions to combat COVID-19 [18]. Regarding human resources, training in telehealth and medical informatics is gradually being introduced in some partner countries, and a diploma in eHealth has been established in partnership with universities in Mali, Senegal, and Ivory Coast [19]. However, the effective implementation of eHealth initiatives remains at a pioneer stage for many countries, with many development paths underway. Some countries have identified the insufficient financing of eHealth, an insufficient number of qualified human health professionals in ICTs (information and communication technologies), and a lack of eHealth priority setting from the ministry of health as barriers to the effective implementation of eHealth initiatives. Despite a global agenda for action in eHealth, the scope of deployment of eHealth initiatives at the country level, aligned with national health priorities, is still relatively low. According to the WHO, only 6.6% of West African countries have effectively implemented a digital health strategy [14]. While many eHealth projects have been developed in sub-Saharan Africa, few have been scaled up due to poor adherence and low uptake rates.

The existing literature highlights various conceptual frameworks that researchers can use to understand potential contextual factors involved in CDSS implementation and classify them into broad axes or domains. These frameworks include the technology acceptance model, the resource-based theory, and the Consolidated Framework for Implementation Research (CFIR) [20]. Although their approaches differ, they all propose a classification of factors that can be useful and adaptable to each implementation project. Overall, the axes integrate the organization or environment in which the CDSS is implemented, the individual behaviors related to the prescriber or patient, and the CDSS itself and its functionalities. Within these broad axes, there are numerous factors, such as practitioner acceptance of new technology, integration of the CDSS into workflows, and access to technical support [21-23]. These factors guide the analysis of potential barriers to CDSS implementation. Many challenges are cited in the literature, such as the poor acceptance of behavior change, the lack of CDSS integration into clinical workflows, the loss of autonomy experienced by the clinician, or the fragmentation of initiatives [23].

However, there is limited literature that focuses specifically on the contexts of the West African countries, as most studies have centered on projects developed in Europe or North America [14,24]. In addition, there is a lack of preimplementation studies that assess microlevel and macrolevel influences, which could be crucial in strengthening the sustainability of eHealth projects by bridging the gap between the digital solution and the reality on the ground. Given the weight of socioeconomic factors in the misuse of antimicrobials, multidimensional studies in this area are underexplored and urgently needed [25-27]. Cross-cultural research that brings together international research centers is also encouraged to overcome these barriers [24].

### Objectives

The main objective of the study was to identify contextual barriers or facilitators directly or indirectly affecting the co-design and implementation of a CDSS for antimicrobial prescribing in 5 sub-Saharan countries (ie, Ivory Coast, Burkina Faso, Mali, Gabon, and Senegal). To anticipate its concrete implementation among health care professionals, the secondary objectives were to analyze prospective implementation strategies, to specify which prescribers and health care structures should be targeted by the CDSS, and to find ways to ensure the sustainability of the CDSS.

## Methods

### Study Design and Participants

This study adopted a qualitative approach through the use of semistructured interviews. The study is reported in accordance with the Consolidated Criteria for Reporting Qualitative Research (COREQ) checklist (Multimedia Appendix 1) [28]. The objective of the recruitment process was to provide a comprehensive understanding of the challenges faced during the development and implementation phases of the CDSS by ensuring a variability of approaches and perspectives. Purposive sampling targeted stakeholders, health actors, scientists, physicians, biologists, anthropologists, project managers, and funders with expertise in antimicrobial stewardship. To this aim, potential participants were identified through a large transnational capacity-building hospital preparedness network in the context of the operational response to COVID-19 in 5 sub-Saharan African countries: Senegal, Gabon, Ivory Coast, Burkina Faso, and Mali [29]. All professionals participating in the network and having a practical or professional experience in the field of antimicrobial stewardship were contacted by email and invited to participate in the study. A reminder email was sent 2 weeks after the first email. Among the 43 potential participants invited to participate voluntarily in a videoconference interview, 21 (49%) answered positively. The individuals who received an email but did not participate in the study either never responded to the contact email or responded once to the email but did not confirm their participation when an interview date was suggested. None of the participants who were interviewed dropped out of the study. The interviews were conducted in French using the Zoom (Zoom Video Communications, Inc) videoconferencing application between September and December 2020. The choice of semistructured interviews appeared more suitable than a stakeholder roundtable for various reasons. First, there existed potential professional interactions among some interviewees as well as hierarchical relationships. These hierarchical relationships hold significant importance and could have led to self-censorship among participants. Second, besides hierarchical relationships, participants worked in different contexts and organizations that do not always collaborate together, and we think there might have been less freedom of expression regarding health system functioning or field experiences. Third, organizing roundtable discussions during the COVID-19 pandemic was judged to be more complicated.

### Interview Schedule

A multidisciplinary team, including infectious diseases clinicians (NP-S and CA), an implementation sciences methodology expert (GB), and eHealth experts in West Africa (EC, SD, and AN), co-designed the qualitative interview guide (Multimedia Appendix 2) based on constructs derived from the CFIR, such as assessing context and needs, local conditions, partnership and connections, available resources, and implementation facilitators [20]. The interview guide was pretested with 3 participants to verify the formulation and the sufficient degree of openness of the questions, as well as the participants’ understanding. All one-to-one interviews were carried out by the same qualitative researcher (EC, a female researcher with a Master of Science degree who was in charge of cross-country coordination for operational and research activities at the time) using a similar approach and began with an introductory presentation of the Antibioclic Afrique CDSS prototype, which was not available for clinicians at the time but already operational and presentable to participants. A simulation demonstrating the use of the tool was presented to them as though they were future users of the tool. This entailed selecting specific decision trees for diseases to ensure they understood how the CDSS could be used. After this presentation, the interviews were aimed at discussing the challenges around antimicrobial prescription, including the barriers to and facilitators of the use of the CDSS, and at identifying tailored implementation strategies.

After identifying the barriers and facilitators according to the CFIR conceptual framework, participants were asked to identify implementation strategies that could help implement Antibioclic Afrique. These strategies were then classified according to the Expert Recommendation for Implementing Change (ERIC) framework, which is a compilation of 73 implementation strategies [30]. These implementation strategies are tailored to address barriers identified with the CFIR framework [31]. The process of linking strategies proposed by the participants to ERIC implementation strategies was continuous and involved discussion and validation during steering committee and staff meetings, as well as identification and debate during individual interviews. On average, the interviews lasted from 45 minutes to 1 hour. Data saturation was collectively discussed after 18 interviews. Participants, while providing specific insights at times depending on their position or location, generally expressed themselves homogeneously on the topics discussed. A total of 3 additional interviews were conducted before closing the interview phase.

### Data Analysis

The interviews were transcribed verbatim by a third-party transcription company, and the data were analyzed through a thematic analysis by a researcher (EC). Key categories were identified and developed into themes through coding. Two other authors (SD and AN) independently coded the transcripts, and the 3 researchers collectively reviewed and reached a consensus on the themes through independent coding and group discussion. The research team then reviewed and agreed upon the major and minor themes. No coding software was used. Preliminary findings and the interview guide were sent to participants and discussed to ensure that conclusions were solidified and to avoid bias in data analysis. Participants found that there was coherence between the collected data and the proposed results.

### Ethical Considerations

This project was approved by the Ethical Committee of Côte d’Ivoire (Comité National d’Éthique des Sciences de la Vie et de la Santé, reference 068-20/MSHP/CNESVS-kp) in June 2020 and the Ethical Committee of Burkina Faso (Comité Éthique pour la Recherche en Santé au Burkina Faso, ref 2021-07-163) in July 2021. No compensation was offered to participants. The research protocol was approved by the Ethics Committee in Infectiology in France (CER-MIT), and a research declaration was obtained from the CNIL (Commission Nationale de l’Informatique et des Libertés). All participants signed informed consent forms before the interviews and received a preinterview information sheet. No personal identifiable information was recorded, and all data were analyzed anonymously.

## Results

### Sample Characteristics

Of the 43 professionals who were contacted, a total of 21 (49%) individuals participated in one-to-one interviews. These 21 participants comprised 12 (57%) men and 9 (43%) women who were working in Ivory Coast (n=3, 14%), Burkina Faso (n=3, 14%), Gabon (n=4, 19%), Mali (n=1, 5%), or Senegal (n=1, 5%). Some participants were working in multiple countries in West Africa (9/21, 43%) and were employed by West African organizations or international organizations. One-third (7/21, 33%) of the participants worked in university hospitals, and nearly one-third (6/21, 29%) worked in nongovernmental organizations (NGOs) specializing in health care. The remaining participants worked in public health agencies (5/21, 24%), foundations (2/21, 10%), and dispensaries (1/21, 5%). Most participants (12/21, 57%) were physicians, while others were health actors, such as technical coordinators (2/21, 10%), project managers (3/21, 14%), experts on antimicrobial resistance (AMR) research (2/21, 10%), a clinical microbiologist (1/21, 5%) and an anthropologist (1/21, 5%). Table 1 provides details on the participants’ characteristics.

**Table 1:** Sociodemographic characteristics of the participants (N=21)^a^.

Characteristics			Values, n (%)
**Gender**			
	Men	12 (57)	
	Women	9 (43)	
**Area of practice**			
	Multiple countries in West Africa	9 (43)	
	Gabon	4 (19)	
	Ivory Coast	3 (14)	
	Burkina Faso	3 (14)	
	Mali	1 (5)	
	Senegal	1 (5)	
**Working structure**			
	University hospitals	7 (33)	
	Nongovernmental organizations	6 (29)	
	Public health agencies	5 (24)	
	Foundations	2 (10)	
	Dispensaries	1 (5)	
**Job title**			
	Physician	12 (57)	
	Project manager	3 (14)	
	AMR^b^ expert	2 (10)	
	Technical coordinator	2 (10)	
	Clinical microbiologist	1 (5)	
	Anthropologist	1 (5)	

^a^Participants in this qualitative study were antimicrobial stewardship experts selected through a transnational capacity-building hospital preparedness network in Senegal, Gabon, Ivory Coast, Burkina Faso, and Mali. Participants had expertise in antimicrobial stewardship and participated in an interview to identify contextual barriers or facilitators directly or indirectly affecting the co-design and implementation of a clinical decision support system for antimicrobial prescribing in their countries.

^b^AMR: antimicrobial resistance.

### Outcomes

#### CDSS Design and Implementation Challenges

**Figure 1:**
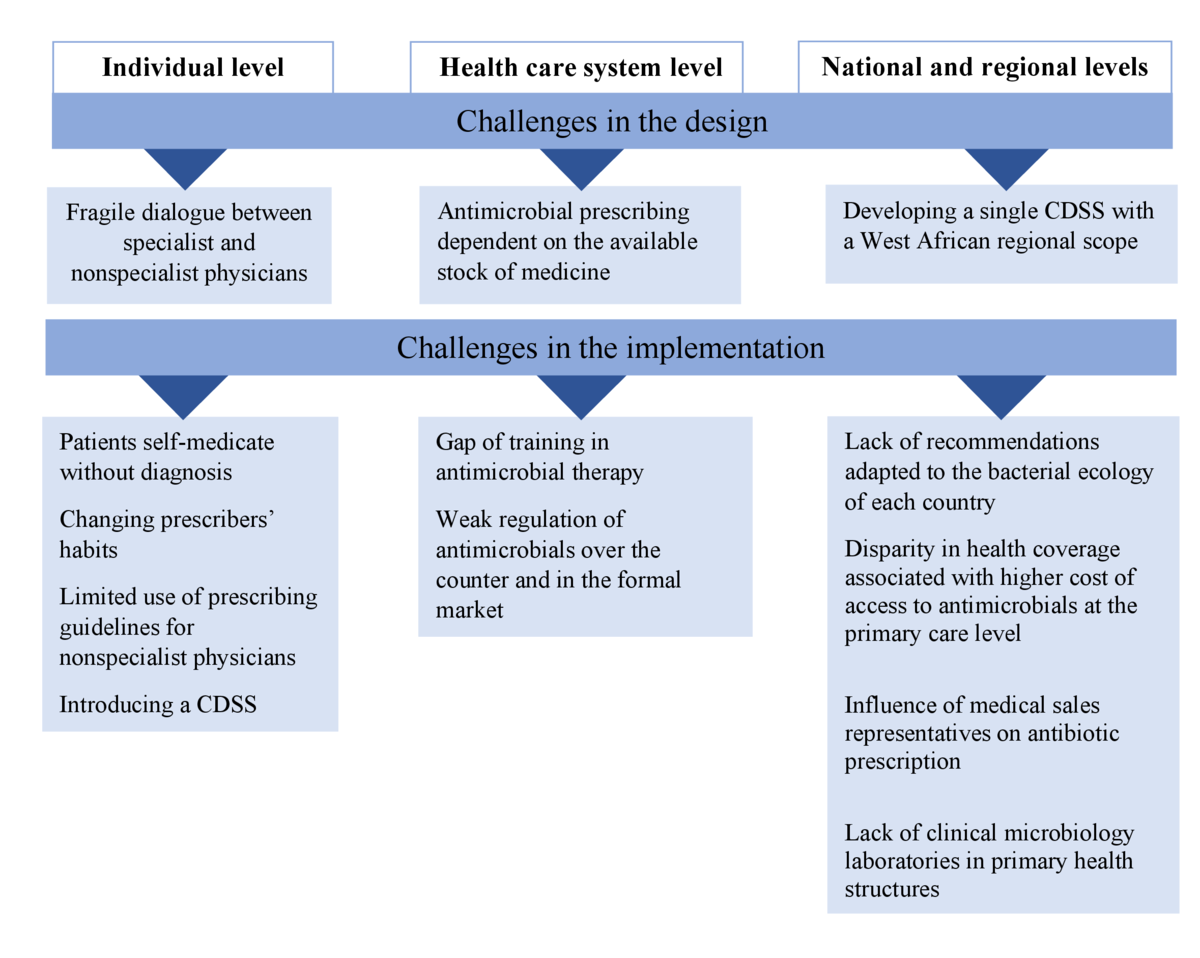
Global challenges in the design and implementation of Antibioclic Afrique. CDSS: clinical decision support system.

The challenges that were identified have been categorized into three distinct levels in Figure 1: (1) the individual level, where the sustainable adoption of the CDSS relies on behavioral patterns and interactions among the public, health care professionals, and patients; (2) the health system level, where factors such as human resource training and the organization of health facilities must be considered to adapt the CDSS; and (3) the regional and country level, where broader political and economic influences that could either facilitate or hinder the implementation of the CDSS must be evaluated.

At the individual level, the participants reported deeply ingrained behaviors and habits both among patients and clinicians regarding the use and misuse of antibiotics, which can pose a significant challenge in moving toward changing practices regarding antimicrobials. The main challenges identified by participants were behavioral factors, such as a lack of common stance in antimicrobial prescribing between specialists and nonspecialists, the belief among many patients in the greater efficacy of antibiotics compared to other types of medications, and the difficulty faced by certain generations of doctors in changing or questioning their professional habits.

Talking about antimicrobial prescribing in clinical practice, a participant noted the following:

As physician-researchers, we don’t have that approach compared to clinicians. Because we are used to seeking guidelines to be sure of what we are doing [with antimicrobials], whereas clinicians, especially the older ones, are not used to looking for references. And I think the problem would not only come from the patients, the sick, but even from the caregivers who could actually not use it [the CDSS] ... Just because it’s part of our daily lives doesn’t mean everyone knows how to prescribe an antibiotic today. People need to accept that they don’t know how to prescribe an antibiotic, that it follows rules, and that these rules are subject to change.

Infectious disease specialist

Talking about the importance of patients’ beliefs associated with the supposed effectiveness of certain treatments such as antibiotics, a participant said the following:

The only thing that will be complicated is the “no antibiotics” approach. Not prescribing antibiotics for the patient should not simply mean “no treatment.” There must be a prescription for a medication. For example, in Casamance where we intervene, traditional medicine is still very much present. We need to consider whether the alternative could be prescribing plants or other remedies, but I believe that patients should leave the consultation with something prescribed. And it’s so strong to the point that, for example, there is a huge placebo effect of the needle, of the infusion. Meaning, you give the same medication, you know perfectly well that the effectiveness is the same, whether it’s intravenous or oral, but in this region, for patients, the symbolism of the needle in the infusion, of the injection, is extremely powerful. It’s the same with antibiotics.

NGO project manager

The participants also identified challenges at the level of the health care system itself, such as the lack of consensus between infectious disease specialists and nonspecialist physicians and the fluctuating availability and cost of certain molecules. The weak regulation of antimicrobials over the counter was also reported to be a variable and may lead to prescribers switching from one class of antimicrobials to another or even not using the CDSS at all.

Talking about the clinicians, a participant expressed the following:

The limiting factor that I see is that if what is recommended is not a molecule available in the central hospital pharmacy or in a pharmacy, or a molecule that is easily accessible, they will not actually prescribe it. And they’ll stick to their current practice of saying, “I’ve got another antibiotic on the side.”

NGO project manager

Talking about the specificity of molecules and its impact on CDSS use by clinicians, a participant said the following:

If your recommendations suggest molecules that are not readily available, they [the prescribers] will actually turn away the second, third, fourth time they are recommended. If it’s not a molecule they can order, they will give up.

In addition to the variability in the availability of antimicrobials, these drugs are also widely available over the counter in pharmacies, allowing patients to self-prescribe without consultation or diagnosis. Some participants noted that the COVID-19 pandemic has exacerbated this issue, with an increase in informal and unregulated suppliers of antimicrobials. This could potentially undermine the impact of CDSSs for antimicrobial prescribing:

The second major prescriber in these regions is the pharmacist because what happens for the patient is that instead of paying for a consultation and then going to the pharmacy, they go directly to the pharmacy. And the pharmacist prescribes, I won’t say for every other patient, but it’s significant. And from what I’ve seen anyway, because it could be the pharmacist or the pharmacist’s assistant. People come to the pharmacy to get their prescription.

NGO project manager

There is also a problem of availability and even cost. There is no Social Security, so it’s the patients who pay. A very illustrative example: when you take an antibiotic like Ceftriaxone which should be the second- or third-line antibiotic, it costs less than one euro compared to the antibiotic like amoxicillin which should be the first line. These are quite a few reasons that can impact on the quality of antibiotic prescribing.

Infectious disease specialist

At the national level, participants identified a key challenge: the absence of national guidelines for prescribing antimicrobials, coupled with a lack of understanding of the local bacterial ecology. They also highlighted the influential role of sales representatives in antimicrobial prescribing:

One of the barriers we see in our context, due to the absence of human resources, is that sometimes there are no recommendations for the prescription of certain antibiotics.

Infectious disease specialist

If you see someone using recommendations for antibiotic treatment, they are French recommendations. For someone who reads a lot, they will seek out American or British recommendations or those from other countries. They will try to compare all recommendations and then draw something from them, in a personal and individual way. So, we don’t have data that are built on the basis of our local ecology. So, probabilistically, in Europe, yes, we could treat a patient arriving at the emergency room with a third-generation cephalosporin as a first-line treatment, because in Europe, the resistance level of E. coli to third-generation cephalosporins is less than 20%. But here, I’m not sure that’s the case. That’s the challenge.

General practitioner

The profile of fevers, both in outpatient and inpatient settings, has changed dramatically in West Africa. I assure you that bacterial infections do not even represent 30% of fevers in hospitals. And even less in outpatient clinics. But antibiotic prescribing, I think it’s over 50% to 60% of prescriptions that include at least one antibiotic.

Infectious disease specialist

Regarding the influence of the private sector in prescribing, a participant said the following:

It is much more the medical representatives who greatly influence the prescriptions of doctors, especially non-infectious disease specialists. They are really influenced by medical representatives, and that is a big problem.

General practitioner

The participants also emphasized the challenge of motivating countries to take further action at the national level for better regulation of prescriptions.

Participants suggested that an eHealth tool could serve as a catalyst for change by incentivizing priority setting in health strengthening policies and promoting regional governance to combat AMR:

Antibioclic Afrique could be a springboard for SAPI [African Society for Infectious Diseases] to initiate a reform on algorithms, protocols and recommendations concerning antibiotic therapy through the appropriation of the tool by different actors. SAPI has a major role to play because the majority of French-speaking African countries are represented in the organization. We must not stop only at our scientific level but go precisely to the level of decision-makers, politicians, regulatory authorities to see how to try to change attitudes and to formalize the algorithms, the recommendations for the management of infectious diseases, the different protocols and ensure that they are applied at the national level by each of the countries.

Infectious disease specialist

#### Identifying Potential Future Users of the CDSS

**Figure 2:**
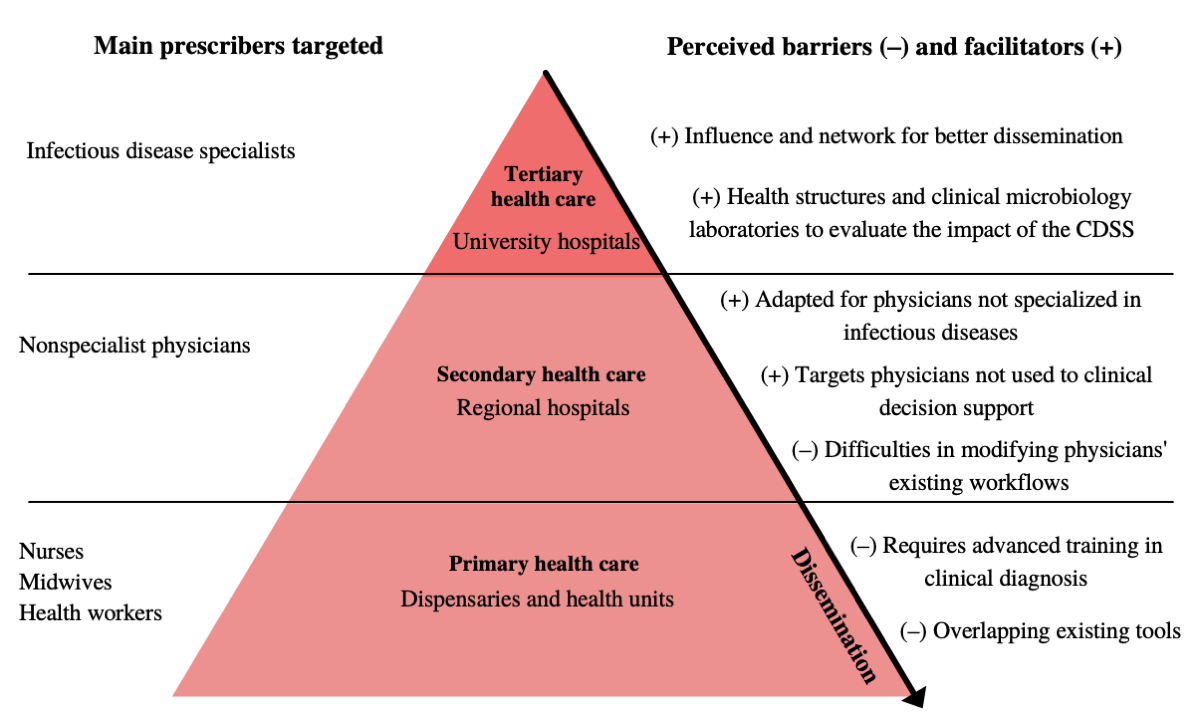
Barriers and facilitators at the different levels of the health pyramid. CDSS: clinical decision support system.

After discussing the general challenges in implementing a CDSS, the participants addressed the issue of its target users. They recognized the need to analyze which health care professionals would benefit the most from the CDSS to support its dissemination and adoption. Figure 2 depicts the barriers and facilitators that need to be considered in CDSS implementation at different levels of care. The organization of health care has been represented as a pyramid of care, with each level corresponding to the number of patients cared for at each level: primary, secondary, and tertiary care. Each level is associated with specific types of physicians (general practitioners or specialized physicians), paramedics (midwives or nurses), or health workers and specific types of health structures [32].

Participants discussed the issue of CDSS target users and shared the assessment that midwives, nurses, and community health workers are key prescribers at the primary health care level:

In Mali, whether officially or unofficially, nurses are sometimes allowed to prescribe medication. ... There are health centers run solely by nurses or often by nursing assistants, which are in distant areas, not close to the city centre.

General practitioner 1

However, most of them stated that the current CDSS architecture was not adapted to their basic training in antimicrobial therapy and clinical diagnosis. The participants identified a major barrier in that a single tool could not be adapted to different types of prescribers with varying levels of training in antimicrobial therapy:

The nurse is trained fundamentally, it’s true, to provide primary care, but they are trained to carry out treatments. The midwife as well. The doctor is trained to analyse a clinical situation and eventually make a decision; they have a diagnostic approach, so the practice is not the same. The tool is great, but it’s essential in its design to consider the user category. It’s easy to say when we’re among ourselves as doctors, but with nurses, with midwives, it needs to be explained well. So, if we’re going to use the tool and make it available to them, it needs to be done from basic training so that they understand the logic of the exercise.

Infectious disease specialist 3

The way the tool is presented, I think it’s more addressed to doctors than to nurses. Nurses in their everyday practice, working with doctors, learn certain things; they start prescribing because they’ve seen doctors do it that way, so they do it that way too.

General practitioner 2

According to participants, the CDSS would be more appropriate for general practitioners, especially in health centers where there are no antimicrobial therapy specialists:

At the prescriber’s profile level, the goal is also to enable individuals who are not specialists in infectious diseases to have sufficient answers, and to have a guide that allows them to recommend or not recommend antibiotics. ... There are antibiotics that normally should not be prescribed by general practitioners, especially imipenem, and it is prescribed haphazardly for any infection without considering antibiotic susceptibility testing. Without any thought, they are prescribed. ... At general hospitals, the likelihood of finding specialists is low; there are mostly general practitioners. There are very few general hospitals where infectious diseases are not encountered. Even the gynaecologist who prescribes an antibiotic behaves like a general practitioner in terms of antibiotic therapy.

Infectious disease specialist 3

Regarding facilitators of CDSS implementation, participants indicated that the initial targets should be nonspecialist physicians in the secondary and tertiary levels of care, where the CDSS could be monitored and better used. Participants also suggested disseminating the tool, starting at the tertiary and secondary levels, so that specialist and nonspecialist physicians can introduce it to their peers in the primary sector and possibly to paramedics. In addition, infectious diseases specialists have an extensive network of connections with public health agencies and health establishments, which can help disseminate the tool:

We know that at the intermediate level, in particular at the level of general hospitals, there is a minimum of technical equipment that already exists, which can be a basis for guiding the decision, unlike at the primary level in rural dispensaries or in health facilities. Because behind a therapeutic decision lies a diagnostic presumption, and even after the treatment has been administered, especially regarding antibiotic therapy, there are monitoring criteria to evaluate effectiveness.

Infectious disease specialist 3

The physician, clinician, researcher, they are always seeking information. ... They will have arguments to encourage their colleagues to adopt the tool. It is necessary to identify healthcare personnel, guide them, train them, whether through webinars or tutorials, and ensure that these trained personnel are willing to transmit the information or knowledge to others, to train other individuals, so that it can be done in cascade.

General practitioner 1

I really think it should be the district chief physician who trains all the district nurses in a single session.

NGO project manager

They also confirmed the need for the CDSS to mitigate antimicrobial misuse in several departments of teaching and regional hospitals:

I was on a test panel last Friday, it was a pilot survey on the use of antibiotics in hospitals, and what came out was that in more than 90% of the departments that were surveyed, an antibiotic had been prescribed, and the main prescribers were in the surgical departments. This to say that this CDSS would come at the right time, because we handle antibiotics, but are we using them correctly?

Infectious disease specialist 3

However, despite these significant challenges, participants were convinced that targeting paramedics as potential regular users of the CDSS is necessary. This will require efforts to adapt the tool and additional implementation work with nurses and midwives, in particular. At this stage, nonspecialist physicians were identified as the primary targets for the CDSS.

#### Implementation Strategies for the CDSS

Given the difficulty in changing primary care prescribers’ routines and the different levels of knowledge in antimicrobial therapy, academic partnerships were cited as one of the most sustainable implementation strategies. A participant suggested that the CDSS could be disseminated through the interuniversity diploma dedicated to antimicrobial therapy [33]:

We already have an Internet site at the level of the University Diploma and we are very willing to disseminate Antibioclic Afrique by popularizing it, already with 150 University Diploma trainees who will each be able to present it to their community.

Infectious disease specialist 2

Even experts contribute to over-prescribing. Antibioclic Afrique will allow us to give them a way of learning, updating existing knowledge and even of applying what they have learned, that is more accessible, according to their context and the context of Android phones, the Internet, etc. Young people like that, so they take it up more easily.

Infectious disease specialist 1

Some participants suggested that creating internal and external networks that connect nonspecialist physicians with antimicrobial therapy experts could be helpful in addressing complex clinical cases and promoting collaborative learning. The dissemination through scientific channels, such as gatherings of the medical community, is also an interesting avenue:

I think that Antibioclic Afrique can really be an opportunity to develop a network of exchanges. We have specialists. We are lucky in Burkina Faso, we have training in antibiotic therapy, practically all the structures have doctors who have been trained here.

Infectious disease specialist 4

Talking about an annual health congress organized in Mali, a participant noted the following:

Utilizing each slot to present the tool is a plus; there are many more specialist doctors, general practitioners, and professors who can also support the transmission of tool usage. They come from all over Mali to attend the conference, so it really allows for a wide dissemination of the message.

General practitioner 1

The participants suggested another measure to support the sustainability of the CDSS, which was to organize continuing education sessions on antimicrobial therapy for health care personnel. Such sessions would not only update their knowledge but also increase the awareness of the risks of AMR for patients and the health care system. These actions would complement the dissemination of the CDSS to potential users and help ensure its long-term viability. Table 2 summarizes the implementation strategies that could be used to promote the adoption of the CDSS according to ERIC categories.

**Table 2:** Identification and classification of implementation strategies suggested during the interviews^a^.

ERIC^b^ strategies	Corresponding specific implementation strategies for the CDSS^c^
Promote network weaving	Develop intrahospital and external networks connecting nonspecialist physicians with trained physicians in antimicrobial prescription
Create a learning collaborative	Identify and train key physicians to develop a network of ambassadors for the CDSS dissemination
Identify early adopters	At the initial phase of implementation, prioritize the intermediary and central levels (university hospitals, general hospitals, and private structures)
Work with educational institutions	Presentation of the CDSS during academic courses for sixth year medical students
Develop academic partnerships	Possible partnership with training degree specialized in antimicrobial prescription
Use an implementation adviser	Experts in implementation sciences involved in the methodology for the CDSS implementation
Use advisory boards and workgroups	Steering committee with representatives of the 5 countries involved
Use advisory boards and workgroups	A total of 21 interviews conducted with health actors to identify implementation strategies of the CDSS
Start a dissemination organization and conduct a local needs assessment	Begin with a pilot site in Ivory Coast, which is responsible for conducting a local needs assessment and disseminating the clinical innovation

^a^Specific implementation strategies for clinical decision support systems (CDSSs) for antimicrobial prescribing in West Africa were identified through interviews with antimicrobial stewardship experts (N=21) and categorized according to the ERIC framework.

^b^ERIC: Expert Recommendations for Implementing Change.

^c^CDSS: clinical decision support system.

## Discussion

### Principal Findings

Participants highlighted the importance of the CDSS for both nonspecialist physicians and primary health care prescribers. They stressed the need for a CDSS that guides nonexpert physicians, given the inappropriate and unregulated antimicrobial prescribing practices observed in various health care settings. Participants also noted that hospitals’ technical platforms could facilitate better monitoring and impact assessment of the CDSS. However, they acknowledged the difficulty in implementing a CDSS that is usable by all primary care prescribers, given their varying levels of training in antimicrobial prescribing.

The main barriers to CDSS implementation cited by the participants were behavioral factors, such as the resistance to changing the physician’s workflow, patients’ self-medication practices, the lack of regulation in antimicrobial dispensing in pharmacies, and the absence of guidelines. To overcome these barriers, participants proposed implementation strategies, such as promoting network weaving, creating a learning collaborative, and identifying early adopters of the CDSS. In addition, they recommended organizing continuing education sessions on antimicrobial therapy for health care personnel to increase the awareness of the risk of AMR and to support the sustainability of the CDSS’s dissemination.

### Comparison With Prior Work

A study conducted in Burkina Faso in 2019 [6] identified the main characteristics required for a CDSS for antimicrobial prescribing, as well as the barriers to and facilitators of its implementation. The study confirmed the importance of a CDSS at the primary care level that is tailored to the local epidemiology of infectious diseases, is available through a smartphone, and includes an offline mode. Co-designing the CDSS with diverse stakeholders was identified as a key strategy to ensure sustainable adoption. Another study found that mobile health (mHealth), a component of eHealth referring to the use of mobile communication technologies to promote health by supporting health care practices, was more accepted and adapted to existing infrastructure and local context due to the rising number of mobile subscriptions in Africa [34].

Beyond a context favorable to the emergence of mHealth tools in LMICs and in Africa more specifically, contextual factors often remain underestimated in the implementation process and need to be identified to optimize successful uptake. Preimplementation studies and holistic evaluations are needed to assess the readiness for eHealth in LMICs, which are still lacking and contribute to the limited uptake of effective clinical innovations [35,36]. Questioning the design of a CDSS necessarily involves understanding the specific context in which it is implemented. Although this study focuses on factors related to the social, economic, and political contexts, such as the level of government involvement in health coverage and health programs, regardless of the country or continent concerned, it is important not to forget other factors. The CFIR conceptual framework, among other conceptual frameworks, offers a useful classification to identify the factors that should be considered to foster the adoption of CDSSs. Factors related to the behavior of patients and health professionals; the functionalities offered by the CDSS; and the specific environment in which it is developed, including the type of infrastructure and level of technology provided, are key elements to evaluate or include in CDSS design [22,23].

In 2014, the Integrated e-Diagnostic Approach (IeDA) was developed and scaled up in Burkina Faso, which is a CDSS for the management of childhood illness at the primary care level. In the preimplementation study of IeDA, participants pointed out that the CDSS must include medicines that are available in the given country and the health care setting. This is necessary to reduce the risk of dropout [37]. In this same project, a multipronged evaluation identified contextual factors that may have been underestimated and limited the implementation of the CDSS. These factors include self-medication, the lack of official guidelines, and difficult access to prescribers [38]. Similarly, ePOCT+ is a clinical decision support system recently developed in Tanzania, Senegal, Rwanda, Kenya, and India for the care of pediatric outpatients in LMIC settings. Its aim is to reduce antimicrobial prescribing. However, 2 limitations of the system are the lack of national guidelines to adapt the algorithms to the local microbial ecology and the difficulty of adherence to the guidelines by practitioners [39]. In Mali, a team is currently developing another CDSS called the Diarrheal Etiology Prediction app. It is available on mobile phones and helps clinicians identify viral childhood diarrhea to reduce antimicrobial prescribing. Preimplementation studies have shown that it is crucial to assess clinicians’ adherence to new technology. This is important to anticipate their hesitation, understand their expectations, and thus promote the sustainability of a CDSS [40]. As existing literature shows, many CDSSs developed in sub-Saharan Africa are addressed to pediatric patients. Conversely, adults, pregnant women, and older adults are underrepresented in algorithms for infectious diseases.

Identifying the health care professionals who will benefit the most from the tool was a crucial aspect of the future CDSS. The results confirmed that, given the gap in training on antimicrobial therapy among health care professionals, the CDSS is more relevant for nonspecialized physicians who mostly work in secondary and tertiary health care facilities. Developing a CDSS in LMICs dedicated to primary health care would require complementary advanced training modules in clinical diagnosis and antimicrobial therapy. Another study conducted on different continents confirmed that financial constraints influence the uneven presence of formally trained practitioners and informally trained practitioners [41]. With regard to behavioral interventions, the same study showed that multifaceted interventions are more effective than educational interventions alone in improving antimicrobial prescribing [41]. Multifaceted interventions that include bundle interventions, regulation enforcement, face-to-face education, and peer influence have the potential to address the behavioral factors cited by the participants.

Some participants who specialize in project management have already developed eHealth tools and shared their best practices and pitfalls to avoid for designing and implementing a CDSS. Other participants shared their global vision of the governance landscape in eHealth and AMR. Regarding the identification of implementation strategies, most of them emphasized the importance of building a solid ecosystem that involves diverse stakeholders from different backgrounds, such as hospital directors, policy makers, associations of health professionals, and the national clinical referent for epidemic management. In the context of the IeDA, a dedicated study recommended designing a stakeholder analysis to tailor the approach and involve each actor in the project [42]. Partnerships with the Health Ministry, health managers, NGOs, and social entrepreneurs were contributing factors to scaling the project. Multisectoral coordination and follow-up, along with incentives for stakeholders, could also be a means to enhance regulatory enforcement for antimicrobial prescription [43,44].

### Limitations

First, most participants in the study were clinicians working at university hospitals. As highlighted by the participants themselves, it would have been interesting to also include nurses, midwives, health workers, and pharmacists who prescribe antimicrobials in primary care in West Africa to provide a broader overview of antimicrobial prescribing. On the one hand, this is explained by the fact that the participants were identified through a network focusing on hospital preparedness. However, these physicians had extensive experience as practitioners, were involved in research projects, and had a global vision on antimicrobial prescribing. On the other hand, although other health care professionals may represent antimicrobial prescribers in primary care in West Africa, the CDSS and its architecture were initially designed for physicians, as the CDSS requires the selection of a specific diagnosis before providing guidance. This explains why recruitment mainly focused on physicians rather than other health care professionals during the preimplementation phase. The CDSS is intended to evolve, and the inclusion of other health care professionals has been considered in the design of future studies. Second, some factors were examined only in 1 country; therefore, their generalizability to the entire western and central African region may be limited. Third, the role of a CDSS for antimicrobial prescribing will highly rely on the existence of local and national antimicrobial prescribing guidelines in West Africa. Indeed, CDSSs such as Antibioclic Afrique may provide targeted recommendations only when guidelines are available. However, the development of guidelines in West Africa is a significant challenge. Fourth, this study may not be generalizable to other CDSSs concerning other diseases or medical specialties. Even though there are some challenges (eg, fragile dialogue between specialist and nonspecialist physicians) and perceived barriers and facilitators (eg, difficulties in modifying physicians’ existing workflows) that might be applicable to the implementation of CDSSs in other medical specialties, this hypothesis would need confirmation studies. Fifth, the thematic analysis of the interviews was conducted by a pluridisciplinary group of qualitative researchers, scientists, and physicians, some of whom were involved in the development of the academic CDSS for antimicrobial prescribing. This might lead to a specific interpretation of the interview data, and their involvement in the design of the CDSS may influence their perception of the obstacles and implementation strategies examined. However, measures have been taken to minimize any potential bias, including ensuring rigorous data analysis and encouraging open and critical discussion among research team members and interview participants. Sixth, this study only examined the use of an expert system to support clinical decision-making based on the guidelines established by the country in which it is used. Evaluating the design and implementation of a machine learning CDSS would require the consideration of other contextual factors, including database management, automatic data entry, and interoperability, which may present significant challenges in LMICs.

### Conclusions

The inappropriate prescribing of antimicrobials and the need for updated knowledge in antimicrobial therapy have been identified as issues that need to be addressed. A CDSS for antimicrobial prescribing might interest West African prescribers. However, significant challenges exist in this context, such as the gap in training for antimicrobial prescribing, antimicrobial prescribers with diverse profiles and levels of expertise, the limited availability of national guidelines, and antimicrobial shortages. A potential implementation strategy for a CDSS for antimicrobial prescribing could be to target physicians in academic hospitals who could then use their network to further disseminate the tool in secondary and primary care. Multilevel partnerships with the government, universities, and medical corporations were identified as crucial means to ensure the sustainable implementation of the CDSS. Further work is needed to explore different contextual factors and settings while addressing the issue of designing one tool for different prescribers, including general practitioners, primary care nurses, midwives, and other health care workers. An upcoming study focusing on user feedback in pilot sites will deepen these results with a focused approach on primary level actors.

## Appendix

Multimedia Appendix 1 Consolidated Criteria for Reporting Qualitative Research (COREQ) checklist.

Multimedia Appendix 2 Interview guide.

## Abbreviations

AMR: antimicrobial resistance
CDSS: clinical decision support system
CFIR: Consolidated Framework for Implementation Research
CNIL: Commission Nationale de l’Informatique et des Libertés
COREQ: Consolidated Criteria for Reporting Qualitative Research
ERIC: Expert Recommendations for Implementing Change
ICT: information and communication technology
IeDA: Integrated e-Diagnostic Approach
LMIC: low- and middle-income country
mHealth: mobile health
SAPI: African Society for Infectious Diseases
SPILF: French Society for Infectious Diseases
WHO: World Health Organization

## Data Availability

The data sets generated during and/or analyzed during this study are available from the corresponding author on reasonable request. Full transcripts of the interview are available upon request (in French).
