# Supplementary material for: Implementation of a Clinical Decision Support System for Antimicrobial Prescribing in Sub-Saharan Africa: Multisectoral Qualitative Study
Source: J Med Internet Res. 2024 Oct 7;26:e45122. doi: 10.2196/45122 (PMC11494254; doi:10.2196/45122)
Supplement: Multimedia Appendix 2 [file jmir_v26i1e45122_app2.docx]

**Multimedia Appendix: Interview guide**

General questions

1) Can you tell us about your job and your function?

2) Did you face any particular difficulties during the COVID-19 crisis? (Lack of equipment, understaffing, lack of coordination). In your opinion, what strategies could facilitate the management of COVID-19 in peripheral health structures?

3) In general, which strategies would you recommend to ensure a successful implementation of Antibioclic Afrique? To your knowledge, has a project of this type already been implemented and what have been the lessons learned?

4) What difficulties do you think we are likely to encounter?

Implementation

1) In your opinion, what are the human and financial resources needed for a project of this type?

2) Do you think that informing and involving influential local public health figures in the dissemination of the tool can facilitate the implementation of the project? Can you mention it?

3) Do you know any experts in E-health who could facilitate the implementation of the project?

4) In your opinion, what educational material could be put in place to ensure that healthcare staff adopt the tool in a sustainable manner in their daily care practice?

5) Through which measures do you think you could adapt the tool to the patient's needs and integrate it into the consultation?

6) In your opinion, which strategies are relevant to disseminate the CDSS?

7) In your opinion, who are the target prescribers and healthcare structures?

International cooperation

7) Which actors should be involved in your country for a project of this type?

8) Are you aware of any other E-health funding that could support ANRS funding?

New technologies

9) Do you know if healthcare professionals are equipped with Android or iOS mobile phones?

10) What are the main barriers to the introduction of new technologies in health in your country?

11) Do you think that the use of an electronic clinical decision support tool can compromise the patient/caregiver relationship?

12) In your opinion, what measures can be taken to raise patient awareness of the use of new technologies in health and in particular of Antibioclic Afrique?

Legal dimension

13) In your country, is there a legislative framework defining scientific research policies? The protection of personal data? The framework of E-health?

14) Do you know of any institutions responsible for coordinating health actions and E-health projects in your country?

Political dimension

15) Do you think that the use of an electronic decision support tool is aligned with national health strategies defined by the government?

16) At what scale should the project be addressed in order to be relevant and to optimize its adaptation (local, regional, national)?

17) What media dissemination strategies would you consider relevant to the project? Do you know of any media or networks targeting health workers?
